# Breast Cancer Disparities in African and African-Ancestry Populations: Genetics, Epigenetics, Structural Barriers and Technology-Enabled Solutions

**DOI:** 10.3389/bjbs.2026.16013

**Published:** 2026-02-27

**Authors:** Chika Eze, Rasha Swadi, Kehinde Ross, Vijay Sharma

**Affiliations:** 1 Cellular Pathology, Royal Liverpool University Hospital, University Hospitals of Liverpool Group NHS, Liverpool, United Kingdom; 2 School of Pharmacy and Biomolecular Sciences, Liverpool John Moores University, Liverpool, United Kingdom; 3 Institute for Health Research, Liverpool John Moores University, Liverpool, United Kingdom; 4 Department of Molecular and Cancer Medicine, Institute of Systems, Molecular and Integrative Biology, University of Liverpool, Liverpool, United Kingdom; 5 School of Medicine, Institute of Clinical Sciences, University of Liverpool, Liverpool, United Kingdom

**Keywords:** African ancestry, *BRCA*1 methylation, breast cancer (BC), health disparities, triple-negative breast cancer

## Abstract

Breast cancer remains a leading cause of mortality among women globally, with disproportionately high incidence, aggressive subtypes and poor outcomes in African and African-ancestry populations. While inherited BRCA1/BRCA2 mutations drive hereditary risk, recent evidence highlights the critical role of BRCA1 promoter methylation especially in sporadic and triple-negative breast cancers (TNBC), which disproportionately affect African-descended women. This review synthesises the genetic and epigenetic landscape of breast cancer susceptibility in African and diaspora cohorts, emphasising unique mutation spectra, elevated BRCA1 methylation frequencies and their prognostic/treatment implications. Systemic barriers including limited screening infrastructure, workforce shortages, structural racism, and cultural challenges exacerbate late diagnosis and inequities. We evaluate emerging solutions such as telemedicine, AI-enhanced diagnostics, and mobile platforms, alongside the need for context-specific research and investment to integrate molecular insights with innovative health system interventions. This synthesis underscores the urgency of addressing biological and structural drivers to close breast cancer outcome gaps in Africa and similar low- and middle-income settings.

## SCOPE AND DEFINITIONS: AFRICA, AFRICAN ANCESTRY AND DIASPORA

This review distinguishes between evidence generated in continental African populations, African diaspora populations, and African ancestry cohorts, and specifies when data are derived from low- and middle-income country (LMIC) settings versus high-income countries. Continental African populations are defined as people residing in countries geographically located in Africa, with a particular focus on sub-Saharan Africa where most studies on breast cancer genetics, epigenetics and health systems have been conducted to date. In line with World Bank terminology, LMIC settings refer to countries classified as low- or middle-income economies (including most sub-Saharan African states), which face constrained resources for cancer prevention, diagnosis and treatment. The African diaspora is defined as communities of African origin living outside the African continent, irrespective of current citizenship or nationality, encompassing populations shaped by historic forced migration (e.g. the trans-Atlantic slave trade) and more recent voluntary migration. In contrast, African ancestry cohorts refer to study samples defined primarily by genomic ancestry (for example, individuals with a high proportion of African genetic ancestry recruited in the United States or United Kingdom), often used as a proxy to interrogate disease biology that may be relevant to African-descended populations more broadly. In this manuscript, data derived from sub-Saharan African settings (e.g. Nigeria, South Africa, Tanzania) are treated as Africa-based evidence, whereas findings from African American, Black Caribbean, and other African-descent populations in the US and UK are explicitly signposted as diaspora or African ancestry proxy evidence, used to fill critical data gaps where equivalent Africa-based genomic, epigenetic or implementation studies are currently lacking.

## Introduction

Breast cancer (BC) is the most common cancer among women globally [1]. Western countries report substantially higher disease-free survival and overall survival rates for BC compared to the rest of the world, reflecting global disparities in early detection and treatment [2, 3]. The 5-year overall survival in high-income countries is above 90% [3, 4]. In the UK, 75% of women survive BC for 10 or more years; mortality is projected to fall further [5]. In major LMICs, outcomes are markedly worse with a 5-year overall survival which is ∼66% in India and just 40% in South Africa [6]. The impact of this disease is particularly devastating in LMIC, where women face earlier onset, higher mortality rates, and more aggressive disease compared to other regions [6–8].

The *BRCA* genes, specifically *BRCA1* and *BRCA2*, encode proteins that are essential for maintaining genomic stability through their critical role in DNA repair. These proteins primarily facilitate homologous recombination (HR), a high-fidelity repair process that uses a homologous DNA template to accurately repair double strand breaks during the S and G2 phases of the cell cycle [9]. The *BRCA1* protein functions as a key regulator by recruiting and interacting with DNA repair factors such as *RAD51* and *PALB2*, promoting the assembly of the repair machinery at sites of DNA damage [9, 10]. Meanwhile, *BRCA2* directly assists in *RAD51* loading onto single-stranded DNA to facilitate homology search and strand invasion, essential steps in HR [9]. Mutations in these genes compromise DNA repair efficiency, leading to genomic instability and increased susceptibility to cancers, particularly breast and ovarian cancer [11, 12].

Inherited *BRCA*1/2 mutations (*BRAC*m) have been associated with increased risk of disease, with *BRCA*m carriers having an approximately 70% cumulative risk of developing BC by 80 years of age [13]. *BRCA*1/2 mutations are found in 5%–10% of all breast cancers, especially in familial cases [14]. However, it is not clear if BRACm patients have worse clinical outcomes than their non-*BRAC*m counterparts [15]. *BRCA* methylation is more common in sporadic (non-hereditary) BC and is particularly prevalent in triple-negative breast cancer (TNBC) which is a more aggressive and faster-growing BC [16–18]. *BRCA*m typically result in loss of protein function due to structural changes that disrupt normal *BRCA* protein activity, rather than a loss of protein expression itself; in contrast, promoter hypermethylation leads to silencing and reduced expression of the *BRCA* gene, which can mimic the effect of a mutation on functional loss by preventing protein production [17, 19].

*BRCA1* promoter hypermethylation is distinct from inherited *BRCA1* mutations but is frequently observed in tumours with TNBC characteristics [20]. The methylation increases cancer risk due to impaired homologous recombination DNA repair [17, 20]. This hypermethylation increases risk of epigenetic silencing and has been noted in both malignant and histologically normal adjacent breast tissues, suggesting its role as an early marker of genomic instability in breast cancer development [19, 21]. While the molecular drivers are still under research, promoter hypermethylation is often considered a primary causative alteration leading to *BRCA* gene silencing in sporadic breast cancers, rather than a consequence of mutated *BRCA* itself [17, 19]. Hypermethylation of the promoter region of the *BRCA1* promoter region, effectively silences its expression without altering the underlying DNA sequence [22, 23]. Both methylation and mutations mechanisms result in defective DNA repair.

This article provides an exploration of *BRCA1* mutation and methylation, its clinical and social implications, and the promise of technology and research investment in closing the BC outcome gap in Africa and other developing regions. We also discuss risk-reducing measures, the importance of breast screening, and the unique challenges and opportunities for managing ductal carcinoma *in situ* (DCIS) in these populations.

## Methodology

This narrative review synthesised evidence on breast cancer disparities in African populations through targeted searches of PubMed, Embase, Scopus and Web of Science databases, covering publications from 1980 to January 2026 to capture foundational genetic studies alongside emerging epigenetic and implementation research. Key search terms included combinations of “breast cancer” or “BC”, “*BRCA1* methylation” or “*BRCA2* mutations”, “triple-negative breast cancer” or “TNBC”, “Africa” or “sub-Saharan Africa” or “African ancestry” or “African diaspora”, “LMIC” or “low- and middle-income countries”, and “epigenetics” or “genomics” or “health disparities.” Inclusion criteria prioritised peer-reviewed human studies reporting primary data from tumour tissues (e.g., promoter methylation) over blood-based germline analyses, excluding animal models, case reports without quantitative data, and non-English publications; studies were required to specify African or African-ancestry cohorts for relevance. Evidence was prioritised hierarchically by study design, favouring systematic reviews and meta-analyses (e.g., on survival gaps or screening barriers), followed by prospective cohorts (e.g., the African Breast Cancer—Disparities in Outcomes [ABC-DO] study), case-control genetics studies, and high-quality cross-sectional reports, with quality assessed via narrative synthesis and tools like the mixed methods appraisal tool (MMAT) where applicable.

## Triple-Negative Breast Cancer (TNBC): A Brief Introduction

Epigenetic changes in BC susceptibility genes are key factors in shaping cancer risk, prognosis, and treatment response. TNBC, an aggressive subtype lacking oestrogen receptor, progesterone receptor and HER2 expression disproportionately affects African American women, particularly younger individuals, with incidence rates nearly twice those observed in White American women [24, 25] (Table 1). These disparities partly reflect unique genetic and epigenetic profiles in TNBC tumours from African American women, including distinct DNA methylation patterns and gene expression changes (e.g., androgen receptor downregulation) enriched in hormone signalling, muscle function and proliferation pathways [26]. Such modifications contribute to heightened aggressiveness and basal-like phenotypes, highlighting the need for tailored therapies [26, 27].

**TABLE 1:** Summary of the epigenetic landscape in TNBC tumours of African American women and their distinct modifications [26, 27].

Epigenetic modification description	Implications
Distinct DNA methylation profile	Alters gene expression, particularly in hormone, muscle, and proliferation pathways
Gene expression changes	Includes downregulation of androgen receptor (AR), influencing tumour aggressiveness
Epigenetic changes enriched in hormone signalling, muscle function, and cell proliferation pathways	May contribute to increased tumour aggressiveness and basal-like phenotype
Differential expression linked to methylation changes, creating a unique molecular subtype	Suggests TNBC in younger black women is epigenetically and clinically distinct
Aberrant histone deacetylation and miRNA dysregulation affecting gene silencing and activation	Represent potential therapeutic targets using HDAC inhibitors and miRNA modulation

TNBC disproportionately affects women of African descent, with African ancestry cohorts showing 10.9% *BRCA1/RAD51C* methylation prevalence versus 4.2% overall linked to higher TNBC rates and poorer prognosis [28]. These biological differences, including distinct epigenetic profiles enriched in hormone signalling, muscle function and proliferation pathways, alongside socioeconomic and systemic factors such as healthcare access barriers and structural racism, contribute to the elevated prevalence and worse outcomes observed in Black women with TNBC [24, 26, 28].

## The Biological Drivers of Disparity Both Genetic and Epigenetic in *BRCA*

*BRCA1* gene methylation plays a significant role in BC development among Black women, contributing to aggressive tumour biology and poorer outcomes [29]. DNA methylation commonly occurs at CpG islands within the promoter region, leading to reduced transcription and loss of *BRCA1* protein function [30]. This process involves the action of DNA methyltransferases (mainly DNMT3a and DNMT3b for *de novo* methylation), which add methyl groups to cytosine residues, especially in the promoter region of *BRCA1* [31]. While *BRCA1* has been extensively studied, emerging evidence shows that *BRCA2* mutations are also prevalent in African American women, with a reported frequency that in some cohorts surpasses *BRCA1* mutations, highlighting its importance in hereditary BC risk within this population [32]. The other major BC susceptibility genes such as *ATM, CHEK2,* and *PALB2* also contribute meaningfully to hereditary risk, though their roles are less well characterised specifically in African American women. For example, *PALB2* mutations have been identified in African-descended populations, accounting for a small proportion of BC cases [33]. *CHEK2* mutations appear less frequent in African American women compared to white populations, while *ATM* mutations have a variable prevalence but are recognised as moderate-risk factors across ethnicities [32, 34]. Understanding the combined impact of these genes, is crucial for refining risk assessment and tailoring prevention strategies in Black women, among whom BC mortality rates remain disproportionately high [20, 29, 35].

Recent studies have shown that *BRCA1* or *RAD51C* methylation is present in about 10.9% of BC cases among African ancestry patients, compared to only 4.2% in the general population [28] (Table 2).

**TABLE 2:** Comparing differential methylation frequencies of breast cancer susceptibility genes including *BRCA1* and *RAD51C* in patients of African ancestry versus the general population, based on a recent study [28].

Gene	Methylation frequency in African ancestry BC cases	Methylation frequency in general BC population
*BRCA1*	Included in combined figure of 10.9%	Included in combined figure of 4.2%
*RAD51C*	Included in combined figure of 10.9%	Included in combined figure of 4.2%
*BRCA2*	Rare methylation observed	Rare methylation observed
*BRIP1*	Mostly absent or very low methylation	Mostly absent or very low methylation
*PALB2*	Not notably methylated	Not notably methylated
*XRCC3*	Methylation ≤25% median, generally absent	Methylation ≤25% median, generally absent

## Methodological Considerations in *BRCA1* Methylation

Critical appraisal of *BRCA1* promoter methylation evidence reveals substantial heterogeneity that complicates clinical interpretation and therapeutic decision-making [21, 36]. A primary distinction lies between tumour-specific somatic methylation predominantly assessed in primary breast cancer tissues via methods like methylation-specific PCR (MSP), pyrosequencing, or genome-wide arrays and rare constitutional methylation detectable in peripheral blood or germline DNA, which carries distinct prognostic implications but limited relevance to sporadic cases prevalent in African cohorts [37, 38] (Table 3). Assay variability further confounds results, as MSP’s binary output and arbitrary cut-offs (often 1%–10% methylation) overestimate prevalence compared to quantitative pyrosequencing or Illumina arrays, which reveal continuous methylation levels and site-specific patterns across the *BRCA1* promoter, with heterogeneity exceeding 70% in meta-analyses [21]. Clonality and spatial heterogeneity pose additional pitfalls, as low tumour purity, stromal contamination, or suboptimal sample handling (e.g., formalin-fixed paraffin-embedded [FFPE] tissues) dilute methylation signals, while intratumoural subclonal methylation may not uniformly predict homologous recombination deficiency (HRD) [39]. Moreover, methylation instability under treatment undermines its reliability as a dynamic biomarker: hypomethylating agents like decitabine can induce demethylation and *BRCA1* re-expression, fostering resistance to PARP inhibitors or platinum chemotherapy, as evidenced by methylation-to-unmethylated conversion in relapsed tumours [40, 41]. Standardisation remains critical, MSP dominates older African studies but underperforms against pyrosequencing for prognostic stratification, while arrays suit epigenome-wide discovery yet fail low-level detection relevant to TNBC [36]. These interpretive challenges necessitate standardised quantitative assays, matched pre/post-treatment sampling, and HRD genomic scarring scores to validate methylation as a robust biomarker in African breast cancer populations.

**TABLE 3:** Comparison of common *BRCA1* promoter methylation assays.

Assay	Principle	Strengths	Limitations	Implications for cross-study comparisons
MSP (methylation-specific PCR)	Bisulfite conversion: Primers specific to methylated/unmethylated DNA	High sensitivity (>1% methylation); rapid; low cost [42]	Binary (positive/negative) output; arbitrary cut-offs; poor reproducibility across labs [21]	Overestimates prevalence; limits quantitative meta-analyses
Pyrosequencing	Bisulfite conversion; quantitative sequencing of individual CpGs	Precise % methylation per site and validated for FFPE [43]	Higher cost; requires specialised equipment; limited regions	Enables dose-response analyses but threshold variability persists
MS-HRM (methylation-sensitive high-resolution melting)	Bisulfite PCR; melting curve analysis	Ultra-sensitive (1% detection); cost-effective for screening [44]	Semi-quantitative; stochastic at low levels; annealing temp-dependent	Superior for low-level constitutional methylation but microarray discordance
Illumina BeadChip (450K/EPIC)	Array hybridisation post-bisulfite; β-values (0–1)	Genome-wide; high-throughput; standardised processing (ChAMP pipeline) [17]	Low sensitivity; underestimates tumour methylation [44]	Population studies miss low-level changes; poor for HRD correlation
NGS (next-generation sequencing)	Bisulfite sequencing; deep coverage	Gold standard resolution; detects subclonality; HRD integration [45]	High cost; bioinformatics intensive; input-dependent	Reference method but rare in African cohorts due to resource constraints

## Prognosis and Treatment

*BRCA1* methylation is associated with poorer overall survival, especially in untreated patients with TNBC [38]. However, it also predicts enhanced sensitivity to DNA-damaging therapies such as platinum-based chemotherapy where tumours with *BRCA1* methylation often respond well to platinum agents, as their impaired DNA repair makes them more susceptible to these drugs [38, 46].

The poly (ADP-ribose) polymerase (PARP) Inhibitors are targeted therapies which exploit the DNA repair defect caused by *BRCA1* methylation, offering promising results like those seen in patients with *BRCAm* [47]. PARP inhibitors function by blocking the catalytic activity of PARP1/2 enzymes, essential for the repair of single-strand DNA breaks through the base excision repair pathway [47]. Inhibition results in accumulation of single-strand breaks, which collapse replication forks during DNA replication, generating lethal double-strand breaks particularly in HR-deficient cells [48]. Additionally, many PARP inhibitors trap PARP enzymes on DNA, further impeding DNA repair and replication fork progression, thereby inducing synthetic lethality in cancer cells deficient in HR repair [48].

Experimental drugs like decitabine aim to reverse methylation and restore *BRCA*1 expression, potentially enhancing the effectiveness of other treatments [49]. However, tumours can adapt by reversing methylation during therapy, restoring *BRCA*1 function and developing resistance [50]. This dynamic nature underscores the need for ongoing monitoring and personalised treatment strategies (Table 4).

**TABLE 4:** Tested drugs to reverse *BRCA*1 methylation.

Drug	Mechanism	Test status	Evidence
5-azacytidine (Vidaza)	DNMT inhibitor, demethylates DNA	Preclinical, clinical trials	Reactivates silenced genes via demethylation [22]
Decitabine (Dacogen)	DNMT inhibitor, hypomethylating agent	Preclinical, clinical trials	Similar mechanism to 5-azacytidine, some clinical activity noted [51]
Hydralazine	Non-nucleoside DNMT inhibitor	Preclinical	Promotes DNA demethylation [52]
Valproic Acid	HDAC inhibitor (indirect effect)	Preclinical, early trials	Histone modification; some demethylating synergy in combination [53]

## Breast Cancer Genetics in Africa

The current landscape of BC genetics in Africa is marked by both progress and persistent gaps. While *BRCA*1 and *BRCA2* mutations are recognised as significant contributors to BC risk in African populations, the spectrum of mutations and their clinical implications can differ markedly from those observed in other regions [54, 55]. In sub-Saharan Africa, studies have highlighted a substantial prevalence of pathogenic variants, reaching up to 20% of inherited invasive BC cases linked to *BRCA1/2* or other DNA repair genes such as *PALB2* and *TP53* [55, 56]. For example, studies in Nigeria have revealed a high frequency of *BRCA1* and *BRCA2* mutations, with up to 20% of inherited invasive BC cases linked to these genes or related DNA repair genes such as *PALB*2 and *TP53* [55]. Similarly, studies in South Africa and East Africa have detected multiple recurrent and novel mutations, with some founder variants identified, such as in the Western Cape population of South Africa [56–58].

The limited number of studies and the focus on a narrow set of genes restrict our understanding of the full genetic landscape, including the role of epigenetic changes like *BRCA1* methylation and the impact of other, less-studied genes. Emerging evidence also points to the importance of epigenetic alterations, notably *BRCA1* methylation, as an additional layer of gene regulation affecting carcinogenesis and treatment response in varying African populations (sub-Saharan African, Middle East and North Africa). While germline mutations have been extensively studied, the clinical and epidemiological impact of gene promoter methylation and other epigenetic mechanisms remains underexplored in these settings and requires further investigation to inform screening and targeted therapies [55, 58].

## Disparities and Challenges

Women of African ancestry are more likely to develop TNBC and other aggressive subtypes, in part due to unique genetic and epigenetic profiles, including higher rates of *BRCA1* methylation [26, 59, 60]. However, most studies on *BRCA* methylation have been conducted in non-African populations, leaving critical gaps in understanding its true prevalence and impact across ethnic groups [55]. A recent mixed-methods systematic review [61] provides a comprehensive analysis of barriers to breast cancer screening among women of Black African and Black Caribbean descent in the UK, with implications highly relevant to African populations globally. The review synthesises qualitative and quantitative evidence, categorising six key barriers: knowledge and understanding, misconceptions about risk, emotional responses, healthcare access, beliefs about treatability, and the influence of religious/cultural factors (Table 5).

**TABLE 5:** Summary of barriers according [61].

Barrier theme	Example/Manifestation
Lack of understanding	Uncertainty about the procedure and reliability of results
Misconceptions and low perceived risk	Underestimating personal risk; attributing cause to non-medical factors
Emotional barriers	Fear of cancer, treatment, partner loss, or stigma
Healthcare access	Negative staff experiences, lack of GP registration, practical inaccessibility
Fatalism about treatability	Seeing cancer as untreatable or inevitable
Religious/cultural factors	Belief in spiritual protection or causes, community taboos, stigma

The review observed that while some interventions (e.g., community education, awareness workshops, telephone reminders) modestly improved screening uptake, few were tailored to Black African or Caribbean women, and no interventions directly addressed emotional, cultural or religious barriers. The authors recommend the development of targeted strategies, greater inclusion of eligible women in studies, and improved cultural competence among healthcare providers [61]. This systematic review [61] demonstrates that barriers to breast screening among women of African descent are multifaceted, spanning knowledge deficits, emotional responses, practical and systemic challenges, and deeply rooted cultural beliefs. Addressing these barriers requires tailored, culturally competent interventions and further research focused on the specific needs of Black African populations.

Women in LMICs, especially in Africa face a confluence of challenges that hinder early diagnosis and effective treatment. These include limited access to screening as many regions lack the infrastructure for routine mammography or molecular testing. The shortage of trained oncologists and pathologists leads to delays in diagnosis and treatment, exacerbating disease progression [62]. There are financial constraints due to high out-of-pocket costs force many patients to abandon treatment or seek alternative therapies [63]. The cultural stigma, lack of awareness, misconceptions and fear of cancer can delay help-seeking and adherence to treatment.

### Structural Racism - A Factor Driving Racial Disparities

Abdelhadi et al. [64]’s systematic review integrated evidence from 29 studies linking multiple indices of structural racism to adverse breast cancer quality of care outcomes, including heightened mortality, later-stage diagnoses, and suboptimal treatment receipt. Structural racism represents a foundational and pervasive factor driving racial disparities in breast cancer outcomes among non-Hispanic Black women, as extensively reviewed by Abdelhadi et al. [64]. Structural racism can be described as the macro-level systems, policies, and processes that embed racial discrimination within key societal domains such as housing, education, healthcare access, economic stability, and neighbourhood environments perpetuating inequities that underpin poorer breast cancer survival.

Among the most salient measures of structural racism are residential segregation and historical redlining, which spatially confine racial minorities to resource-limited neighbourhoods with limited healthcare infrastructure, impairing access to timely screening, diagnosis, and treatment [65]. These effects are compounded by comorbidities and neighbourhood factors, driving complex, multifactorial impacts on patient care and survival disparities [64]. Furthermore, structural racism exacerbates financial and educational barriers, unequal distributions of social capital, and psychological stress, all of which interact to worsen breast cancer prognosis for Black women. Beyond individual-level interventions, tackling breast cancer inequalities requires systemic and multi-sector reforms to dismantle the pervasive effects of structural racism.

## Solutions for Africa and the Developing World Through Harnessing Technology

### Telemedicine in Breast Cancer Care

Telemedicine platforms have emerged as transformative tools in overcoming healthcare access barriers in sub-Saharan Africa, particularly in breast cancer care. It is aimed at addressing critical gaps in breast cancer diagnosis, treatment, and follow-up care in this region where geographic, economic, and infrastructural barriers impede timely access to specialist services [66]. Telemedicine which has been in use since the 1960s is defined as the delivery of healthcare services at a distance using electronic and telecommunication technology for diagnosis, treatment, monitoring, and prevention of diseases and injuries, as well as the education of healthcare providers and patients [67]. These platforms enable local healthcare workers to connect in real-time with remote oncologists and specialists for consultations, diagnostic support, and ongoing training.

**FIGURE 1:**
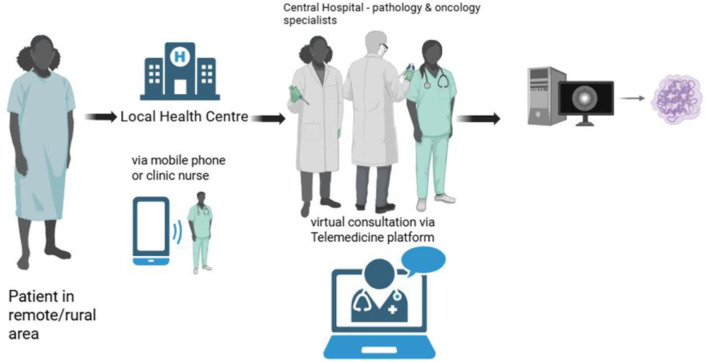
How Telemedicine works by connecting patients and clinicians remotely using digital communication tools. It transmits clinical data, images, and video from a local site to a specialist, who reviews the information, gives advice or a diagnosis, and may prescribe treatment or follow-up, reducing the need for in-person visits. Figure was created by author on BioRender.com.

Historically, telemedicine evolved from basic telephone consultations and isolated specialist outreach to sophisticated digital platforms enabling live multidisciplinary tumour boards or meetings, remote treatment supervision, and patient education through mobile health (mHealth) applications [68]. A leading example is Project ECHO (Extension for Community Healthcare Outcomes), which originated in the United States and has been successfully adapted in South Africa to improve early detection and management of cancer [69]. This model empowers primary care providers through knowledge-sharing networks, mitigating delays traditionally caused by scarce specialist workforce and geographic isolation (Figure 1).

Several studies document telemedicine’s role in enhancing breast cancer outcomes by providing timely access to specialist expertise without the financial and logistical burdens of travel [70, 71]. Tele-oncology services facilitate symptom recognition education, triage, and treatment adherence monitoring while especially benefiting rural patients [71, 72]. Many women, particularly in rural areas, must travel long distances to access care, often at great personal and economic cost [73, 74]. Others may forgo treatment altogether, prioritising their families’ immediate needs over their own health. These stories underscore the urgent need for a holistic, people-centred tele-oncology initiatives in sub-Saharan Africa. Tele-oncology remains relatively scarce; however, the outcomes observed thus far are encouraging and hold substantial promise for the future [75].

Nonetheless, the challenges remain including limited broadband infrastructure, intermittent power supply, poor digital literacy among both providers and patients, and cultural barriers affecting telemedicine acceptance [68]. For example, conservative gender norms and reliance on traditional healers may reduce uptake of telehealth services among women at risk of breast cancer in some regions [68]. Addressing these challenges requires culturally sensitive community engagement, robust infrastructure investment, and training programs to build digital competencies. The prospects include integrating emerging technologies such as artificial intelligence to enhance diagnostic accuracy and personalised care pathways [70]. The ongoing expansion of telemedicine models like ECHO across African nations signals a promising paradigm shift towards democratising cancer care in low-resource settings.

### Digital Platforms and Apps in Breast Cancer Care

Digital platforms and apps are being developed to provide evidence-based information on BC screening and early detection, targeting both patients and frontline health workers [76, 77]. Many women in sub-Sahara Africa live far from tertiary health facilities, and there are gaps in awareness of BC risk factors and symptoms. Mobile apps provide accessible, locally relevant, evidence-based information about breast cancer, self-examination, and the need for early detection directly to users’ phones, even in remote areas where smartphone penetration is rising rapidly (over 86% coverage in some countries like Burkina Faso) [78]. Barro et al. [78] developed an artificial intelligence platform dedicated to breast cancer detection with key features such as AI-powered automated breast cancer image analysis, a major limitation in many African regions. The platform incorporates an automated analysis of histopathology images using an artificial intelligence (AI) model, based on convolutional neural networks to support pathologists by detecting abnormalities, predicting cancer subtypes, and flagging cases that require urgent attention, even where local expertise is scarce [78].

Improved smartphone usability enables pathologists in remote African settings to access advanced digital histopathology tools and patient management features, enhancing diagnostic equity and reach. However, AI models for histopathology are predominantly trained on high-income country datasets lacking African representation, leading to performance disparities and lower accuracy in Black patients for cancer subtyping and risks of bias, misdiagnosis or perpetuated inequities [79, 80]. Limited or patchy access to stable internet, electricity, and mobile data in many rural or peri-urban African settings can hamper real-time uploads, large-image processing, and access to cloud-based AI tools, restricting the utility of the platform. While smartphones are widespread, reliable and secure mobile devices with sufficient processing power may still be beyond reach for some users.

Additionally, there are potential costs associated with maintaining, updating, and supporting the AI tools over time. Other challenges include the integration of AI with existing pathology workflows, medical records, and communication channels which can be complex [70]. Resistance to change, lack of training for local clinicians, or concerns over job displacement could also impact adoption. Issues around patient data privacy, informed consent, and clear regulatory standards for AI use in clinical practice are magnified when digital systems across international borders and diverse legal frameworks [70]. Although the AI-enhanced Android platform can broaden diagnostic access, it must overcome challenges in data representativeness, digital infrastructure, local adaptation, sustained funding, and data governance to achieve equitable and reliable impact in breast cancer care in Africa.

### Breast Screening

Breast screening is a critical tool in the early detection and management of breast cancer, significantly reducing mortality rates in high-income countries [81–84]. However, the African context presents unique challenges that hinder the widespread adoption and effectiveness of breast screening programmes. The reality for many women in Africa is shaped by limited infrastructure, younger age at diagnosis, and significant resource constraints. These factors combine to create a landscape where early detection is often the exception rather than the rule, and where the human cost of BC remains unacceptably high [85, 86]. In much of Africa, organised national breast screening programmes are rare, with only a handful of countries having established such systems. Most women rely on opportunistic screening, which is often inconsistent and inaccessible, particularly for those living in rural or remote areas [85]. The shortage of specialised cancer centres and trained healthcare workers further compounds the problem, leading to late diagnoses and poorer outcomes. For many women, the journey to a healthcare facility is long and costly, and the fear of diagnosis, coupled with limited awareness, can delay help-seeking behaviour [86, 87].

A significant challenge in the African context is the younger age at which many women develop BC [88]. African women are more likely to be diagnosed in their thirties or forties, a time when breast tissue is denser, and mammography is less sensitive. This biological reality means that mammography, the gold standard for screening in older women, may be less effective for a large proportion of the population [85]. Competing health priorities and limited healthcare budgets mean that investment in mammography infrastructure is often deprioritised, leaving many women without access to this potentially life-saving technology [86]. Despite these challenges, innovative approaches are emerging that offer hope for improving early detection. Clinical breast examinations (CBEs), when performed by trained health workers, provide a cost-effective and accessible alternative to mammography, especially in settings where resources are limited. However, uptake of CBEs remains low, with studies highlighting barriers such as financial constraints, long distances to healthcare facilities, and the need for permission to access care [87]. These barriers are particularly acute for women in rural areas, who may face additional challenges such as lack of childcare and competing family responsibilities [86].

At the heart of these efforts are the women whose lives are affected by breast cancer. For many, the barriers to screening and treatment are not just logistical or financial, but deeply personal. The fear of a cancer diagnosis, the stigma associated with the disease, and the competing demands of family and work can all influence whether a woman seeks care. Reports from rural communities in The Gambia, for example, highlight how women with multiple children are less likely to participate in screening, often prioritising their families’ needs over their own health [86]. Conversely, women with formal employment or higher education are more likely to access screening, reflecting the broader social determinants of health that shape cancer outcomes.

### Risk-Reducing Measures

Portable ultrasound devices are another promising tool, enabling health workers to triage palpable lumps and provide diagnostic support in younger women with dense breast tissue. Community outreach initiatives, including mobile screening units and pop-up clinics, are helping to bridge the gap by bringing services directly to women in underserved areas. These initiatives not only improve access but also foster trust and engagement within communities, addressing some of the cultural and social barriers that can impede screening uptake [87]. The diagnosis and management of ductal carcinoma *in situ* (DCIS) in Africa further illustrate the disparities in BC care. DCIS, a non-invasive form of breast cancer, is commonly detected through screening in high-income countries, where it is typically managed with surgery and sometimes radiation, resulting in excellent prognosis. In Africa, however, the lack of widespread screening means that DCIS is rarely identified at an early stage. Most cases present as invasive cancer, by which time treatment is more complex, and outcomes are poorer [85].

Pathology services, essential for the accurate diagnosis of DCIS, are limited in many African countries. Even when DCIS is correctly identified, treatment options may be limited by the availability and affordability of surgery and radiation therapy, leaving many women without access to the care they need [86]. Addressing these challenges requires a multifaceted approach. Strengthening pathology services through investment in training and equipment is essential for improving diagnostic accuracy. Developing and adapting clinical guidelines to reflect local realities can help ensure that care is both evidence-based and feasible within resource-constrained settings. Research into the natural history and outcomes of DCIS in African populations is also crucial, as it will inform best practices and guide the development of context-appropriate interventions [85].

Strengthening health systems is fundamental to improving BC outcomes in Africa, where late diagnosis and limited access to timely, effective care continue to cost thousands of lives each year. The World Health Organisation (WHO) highlighted that without urgent action, an estimated 135,000 women could lose their lives to BC by 2040 in sub-Saharan Africa, a tragedy that not only devastates families but also has far-reaching social and economic consequences [85]. At the heart of this challenge is the pressing need to invest in pathology and laboratory infrastructure, ensuring that women receive timely and accurate diagnoses. Currently, access to pathology services is severely limited, with most countries in the region failing to meet the recommended standard of one laboratory per 100,000 people [85]. This shortfall leads to delays in diagnosis, misclassification of disease, and missed opportunities for early intervention, all of which contribute to high mortality rates.

Addressing the shortage of specialised healthcare professionals is equally crucial. Many African countries face a critical lack of oncologists, surgeons, and pathologists, making it difficult to provide comprehensive cancer care [85, 89]. Task-shifting and upskilling training non-physician health workers to deliver basic cancer care have emerged as practical solutions. By empowering nurses and community health workers to perform clinical breast exams, provide patient education, and support treatment adherence, health systems can extend their reach and free up specialists to focus on complex cases [90, 91]. This approach not only bridges workforce gaps but also brings care closer to the communities most in need, reducing barriers related to distance and cost.

Financial and social support mechanisms are essential to ensure that women can access and complete their treatment. Out-of-pocket costs for diagnostics and therapy remain prohibitive for many families, often resulting in delayed care or abandonment of treatment altogether [92]. Subsidising diagnostics and treatment through government funding or partnerships with non-governmental organisations can significantly reduce these financial burdens. Patient navigation services, which help women overcome logistical barriers such as transportation, accommodation, and childcare, are equally important. These services are not merely administrative; they offer emotional support and advocacy, guiding women through a complex and often intimidating healthcare journey [92]. For many, the difference between life and death can hinge on the presence of a compassionate navigator who ensures they do not fall through the cracks.

Progress is possible, as demonstrated by successful initiatives in countries like Egypt and Algeria, where investments in early detection, workforce training, and comprehensive treatment have led to significant improvements in survival and quality of life [93]. In Egypt, for example, a nationwide screening programme has resulted in increased early detection of BC cases, while also providing a strong economic return on investment [93]. These examples show that, with coordinated action and sustained investment, it is possible to rewrite the reports of BC in Africa by transforming it from one of loss and despair to one of hope and resilience. It is crucial to engage in community-based awareness and education using culturally tailored campaigns. The use of local languages and community leaders to dispel myths and encourage early presentation within their community can encourage engagement. Within the school-based education system, girls and young women can be taught about breast health and self-examination. An important solution will be funding and expanding access to screening. Schemes such as mobile mammography units can bring screening directly to rural and underserved communities. Simultaneously training frontline health workers will empower nurses and community health workers to perform clinical breast exams and refer suspicious cases.

### Genetic Counselling as Standard of Care

Evidence suggests Black women have a higher prevalence of pathogenic variants in breast cancer susceptibility genes, especially in younger patients and TNBC cases, with studies reporting up to a 26% prevalence in key genes such as *BRCA1*, *BRCA2*, *PALB2*, *CHEK2*, *ATM*, and *TP53* among African American women [94]. However, under-referral to genetic counselling and low uptake remain barriers, compounded by issues of medical mistrust, genetic discrimination, and limited availability of genetic counsellors especially in regions with “medical deserts” [94]. There is a compelling argument for offering genetic counselling as a standard of care to African women with breast cancer, stemming from the distinct genetic landscape seen in their tumours, and the longstanding racial disparities in breast cancer mortality and outcomes. Pleasant et al. [94] outline that, while incidence rates for breast cancer are similar between Black and White women, Black women experience a markedly higher mortality around 40% increased risk and poorer stage-specific survival. This is driven largely by a higher prevalence of aggressive subtypes such as triple-negative breast cancer (TNBC) and a greater likelihood of early-onset disease, both of which are themselves indications for genetic counselling and testing under current national guidelines [94].

Current practice in genetic counselling tends to rely on risk-stratified models based on family history and epidemiological data, most of which are derived from studies in White populations. This approach often fails to account for African-specific risk factors and can miss eligible individuals due to inadequate access to family history, incomplete penetrance of known susceptibility genes, and provider bias [94]. Offering genetic counselling to all African women, not just those who meet Eurocentric or family history-based guidelines would facilitate cascade testing among relatives, improve prediction of private variants, and foster equity in precision medicine.

### Investing in Research to Addressing Data Gaps

Most studies on *BRCA* methylation and BC biology have focused on European and North American populations. There is a pressing need for research that reflects the genetic diversity and unique risk factors of African and other LMIC populations [28]. This lack of representation has profound consequences, as it means that diagnostic tools, risk models, and treatment strategies are often less effective or less accessible for African women, who already face disproportionate burdens of late diagnosis and poor outcomes. Data gaps in BC research are not simply a scientific imperative but a matter of equity and justice for women across Africa and other low- and middle-income countries. Building local research capacity is essential to closing these gaps and ensuring that African women benefit from advances in precision medicine [95]. Supporting African-led research through funding, training, and infrastructure is critical. When local scientists are empowered to lead studies, the research questions, methodologies, and interpretations are more likely to reflect the lived realities and priorities of African communities. Establishing regional biobanks and data-sharing platforms can facilitate large-scale studies, enabling researchers to capture the genetic and environmental diversity that shapes BC risk and outcomes across the continent [54]. These resources also foster collaboration and innovation, allowing African researchers to contribute to and benefit from global scientific advances.

Translational research and innovation must be at the heart of these efforts. Developing context-appropriate diagnostics such as molecular tests adapted for use in low-resource settings can dramatically improve early detection and treatment. For example, integrating affordable genetic and epigenetic testing into existing health systems would allow for the identification of high-risk women and families, enabling targeted prevention and surveillance [96]. Representation of African women in clinical trials for new therapies, including targeted and immunotherapies, is equally important. Without such inclusion, the efficacy and safety of these treatments in African populations remain uncertain, perpetuating disparities in care and outcomes. The human dimension of these data gaps is stark. For many African women, a diagnosis of BC comes late, often after the disease has advanced beyond curable stages. The absence of locally relevant research means that clinicians may lack the tools to offer personalised risk assessments or to recommend the most effective treatments. Families are left with uncertainty, and women may feel overlooked by a global research agenda that does not reflect their needs or experiences. Conversely, when research is rooted in local realities, it can empower women and communities, offering hope and agency in the face of a devastating disease.

Jiang et al. [97] addresses critical bioinformatic challenges and solutions to reduce bias against African populations in cancer genomic research. They highlight how most current cancer genomic datasets and bioinformatic workflows are heavily biased towards European ancestry. African ancestral representation remains minimal, largely confined to African American samples, which inadequately capture the rich genetic diversity and unique tumour biology of continental Africans. This bias leads to potential inaccuracies and exclusion of African patients from the benefits of precision oncology. To tackle these challenges, the authors advocate for bioinformatic workflows designed specifically for African-derived datasets. Key solutions include scalable, high-performance computing (HPC) workflows enabling parallel processing of whole-genome sequencing data with high efficiency, thereby managing the substantial data volume and genomic complexity characteristic of African tumours [97]. Their pipeline incorporates physical data chunking and genomic interval “scatter-gather” parallelism strategies to accelerate genome alignment and variant calling steps [97]. Without addressing bioinformatic and resource barriers, African populations risk further exclusion from genomic medicine advances. The authors strongly call for global efforts to allocate resources and develop scalable computational tools adapted to African genetic diversity to reduce disparities in cancer outcomes.

## Telemedicine and AI Prioritisation

Telemedicine via low-cost mobile platforms (e.g., WhatsApp consultations, Project ECHO-style telementoring) offers the most feasible high-impact interventions for sub-Saharan Africa over the next 3–5 years, given the smartphone penetration and proven survival benefits in breast cancer triage [98, 99]. AI pathology tools like Ouattara et al. [100]’s convolutional neural network for histopathology analysis rank lower due to training data biases and infrastructure demands, though portable ultrasound-AI hybrids show promise for dense-breast screening in younger cohorts [100–103].

Implementation barriers include unreliable electricity (124 kWh/capita/year in SSA), patchy 4G connectivity, device maintenance costs, and absent regulatory frameworks, with Nigeria exemplifying power outages and policy fragmentation hindering scale-up [104, 105]. Governance gaps exacerbate data privacy risks under deficient GDPR-equivalent protections, necessitating federated learning models to localise AI training [98]. Cost-effectiveness favours task-shifting via telemedicine, but sustainability demands public-private partnerships for procurement, recurrent costs subsidisation, and continuous provider training to overcome resistance prioritising these yields 2–3x ROI through reduced travel burdens.

## Resource and Cost Gradients Across the Breast Cancer Pathway

Breast cancer control in African settings is constrained by stark gradients in resource availability along the diagnostic and treatment continuum, with pathology, imaging, surgery, systemic therapy and radiotherapy capacity all substantially below international benchmarks. Recent WHO and Global Breast Cancer Initiative–aligned assessments across the WHO African Region show that only a minority of countries have organised breast screening programmes, and just two meet the recommended minimum of one pathology laboratory per 100,000 population, underscoring the structural scarcity of core diagnostic infrastructure [85]. Radiotherapy access is similarly constrained: most sub-Saharan African (SSA) countries fall short of the International Atomic Energy Agency recommendation of one radiotherapy unit per 200,000 people, forcing patients to travel long distances and incur substantial indirect costs [106].

Economic evaluations and health-system analyses further highlight how opportunity costs shape service configuration (Table 6). Government health expenditure *per capita* in many SSA countries remains a fraction of that in North African or high-income settings, limiting the fiscal space for capital-intensive technologies such as digital mammography, linear accelerators, and genomic or epigenomic testing. For example, Limenih et al. [107] report government health spending *per capita* of 17–93 USD in several SSA countries, compared with over 200 USD in some North African states, paralleling lower survival and poorer access to multimodality care. Within this constrained envelope, relatively low-cost interventions such as clinical breast examination (CBE), basic ultrasound and manual immunohistochemistry (IHC) are more feasible to scale than routine mammographic screening or comprehensive genomic panels, which require substantial upfront and recurrent investment.

**TABLE 6:** Indicative resource and cost gradients for key components of the breast cancer pathway in African settings.

Pathway step	Example services	Typical cost band	Typical availability in sub-saharan Africa
Awareness and early detection	Community awareness campaigns; breast health education; clinical breast examination (CBE) in primary care	Low: Mainly staff time, basic training, IEC materials	Variable but potentially scalable; CBE feasible in most primary-care settings if staff are trained and supported [85, 108, 109]
Diagnostic imaging	Diagnostic ultrasound for palpable lumps; opportunistic mammography in urban centres	Medium: ultrasound lower cost than mammography; mammography units and maintenance relatively expensive for many LMIC budgets	Ultrasound available in many secondary/tertiary facilities; dedicated mammography largely limited to major urban centres and private/tertiary hospitals [85, 109]
Tissue diagnosis and basic pathology	Core needle or surgical biopsy; histopathology; basic ER/PR/HER2 IHC	Medium: recurrent costs for consumables, reagents, equipment maintenance; capital costs for laboratory set-up	Severe workforce and infrastructure gaps; many countries below recommended 1 pathology lab per 100,000 population; IHC often centralised in a few reference-centres [85, 110]
Staging and locoregional treatment	Surgery (mastectomy/breast-conserving surgery); basic imaging for staging	Medium: requires theatre time, anaesthesia, peri-operative care; lower capital cost than radiotherapy or genomics	Surgical capacity present in most tertiary hospitals but constrained by limited theatre time, workforce shortages and competing surgical priorities [107, 108]
Radiotherapy	External-beam radiotherapy (often cobalt or linear accelerator); palliative and curative regimens	High: substantial capital outlay, maintenance, shielding, stable power, specialist workforce	Markedly limited; many SSA countries have no radiotherapy units at all; where present, machines are few and often overburdened, with long travel distances for patients [85, 106]
Systemic therapy (standard)	Endocrine therapy; cytotoxic chemotherapy with standard regimens; basic anti-emetics and supportive care	Medium–High: drug and supportive-care costs significant relative to budgets; ongoing supply chains needed	Available in most tertiary centres, but affordability, stock-outs and limited supportive care constrain consistent access; many patients pay substantial out-of-pocket costs [107, 108]
Targeted and high-cost systemic therapy	Trastuzumab and other HER2-targeted agents; CDK4/6 and PARP inhibitors	High – drug acquisition costs often exceed per-capita health expenditure in many LMICs	Very limited outside a small number of tertiary or private centres; often accessible only via special funding, philanthropy or clinical trials [108, 111, 112]
Genomic and epigenomic testing	Germline BRCA1/2 testing; multigene panels; tumour sequencing; BRCA1 promoter methylation assays	High – requires advanced laboratory platforms, specialist staff, bioinformatics, quality systems	Rare and highly centralised; typically, available only through research collaborations, private laboratories or export of samples to high-income countries [110, 113]

## Conclusion

Addressing breast cancer (BC) disparities in Africa, the African diaspora, and other low- and middle-income countries (LMICs) represents both a scientific imperative and a profound human challenge. Integrating genetic and epigenetic insights such as BRCA1 promoter hypermethylation with context-appropriate technological solutions offers a pathway to precision oncology within resource-constrained systems. Progress demands interconnected actions centred on women’s lived experiences and community priorities across continental, diaspora, and ancestry-defined populations.

Key priorities include scaling rapid, low-cost diagnostics and screening modalities feasible in primary-care settings, such as clinical breast examination and ultrasound triage. Strengthening telemedicine infrastructure and continuous professional development for healthcare workers extends specialist expertise to remote and underserved regions, mitigating urban-rural divides. Deploying mobile health platforms for patient education, adherence support, and navigation further empowers women to engage proactively with care pathways. Sustained investment in local research capacity, equitable technology transfer, and health-system strengthening will ultimately bridge outcome gaps, delivering innovation, compassion, and equity to transform BC care globally.

### Limitations

A key limitation of this review is the reliance on published literature, which may under-represent data from low- and middle-income countries and studies not indexed in major databases.
